# Unique Role of Med8 in Ace2 Recruitment and Target Gene Expression in *Schizosaccharomyces pombe*

**DOI:** 10.4014/jmb.2507.07055

**Published:** 2025-10-14

**Authors:** Ji-Hyun Kim, Kyoung-Dong Kim

**Affiliations:** Department of Systems Biotechnology, Chung-Ang University, Anseong 17456, Republic of Korea

**Keywords:** Mediator, Ace2, *Schizosaccharomyces pombe*, mitosis, RNA polymerase II

## Abstract

The Mediator, an essential RNA polymerase II coactivator, is a conserved multi-subunit protein complex present in organisms ranging from yeast to humans. Although its role in transcription is well-characterized, the distinct functions of its subunits remain largely unclear. In this study, we aimed to investigate the roles of Med8, Med14, and Med17 in Ace2-dependent transcriptional regulation in *Schizosaccharomyces pombe*. Transcriptome analysis revealed that depletion of Med14 and Med17 caused widespread transcriptional repression, consistent with their structural roles in maintaining Mediator integrity. In contrast, depletion of Med8 specifically impaired the transcription of Ace2 target genes. Chromatin-binding analysis revealed that despite stable Ace2 protein levels, Med8 depletion led to a remarkable decrease in Ace2 occupancy at the target promoters, indicating that Med8 plays a crucial role in the recruitment of Ace2. In contrast, the binding of Ace2 was largely unaffected by the depletion of Med14 or Med17, underscoring their involvement in the transcriptional activation steps following Ace2 recruitment. Moreover, depletion of Med8 did not influence the expression or genome binding of Med14 and Med17, suggesting that Med8 plays a distinct role, rather than maintaining Mediator stability. Co-immunoprecipitation further revealed a physical association between Med8 and Ace2, suggesting that Med8 may be involved in Ace2-dependent transcriptional regulation. Overall, our findings highlighted that Med8 uniquely promotes Ace2-dependent transcriptional initiation by enhancing the recruitment of transcription factors. The study could advance our understanding of how individual Mediator subunits fine-tune gene expression through direct interactions with specific transcription factors.

## Introduction

The Mediator complex, a crucial protein complex in eukaryotic transcription, performs several basal regulatory functions that extend beyond simple coactivator roles. It acts as a central orchestrator in preinitiation complex (PIC) assembly, coordinating the sequential recruitment and stabilization of general transcription factors, such as TFIIB, TFIIH, TBP, and TFIID, through extensive interactions [1, 2]. Beyond its general regulatory functions, the Mediator plays a critical role in orchestrating cell cycle progression, including mitotic entry, through the periodic transcription of gene clusters [3-5]. It can periodically bind to mitotic clustering genes, ensuring their timely expression, which is crucial for the accurate progression of the cell cycle. The intimate connection between the Mediator and cell cycle regulation serves as the foundation for understanding its specific roles in processes such as cell separation, which is a critical event in the cell cycle.

In the fission yeast *Schizosaccharomyces pombe*, the Mediator complex comprises 19 subunits, including 15 core and 4 Cdk8 module subunits [6-8]. The Mediator in *S. pombe* plays a global role in transcription, with individual subunits contributing to distinct structural and regulatory functions. Med8 is an essential subunit of the head module and is critical for organizing the head subcomplex through interaction with Med18 and Med20 [7, 9, 10]. Moreover, Med8 facilitates the transmission of regulatory signals to RNA polymerase II, partly through interactions with the Rpb4 subunit, to coordinate the expression of genes required for proper cell cleavage and separation [11, 12]. Another essential subunit, Med14, functions as the central architectural scaffold for the Mediator complex. It is the only known subunit that directly interfaces with all three core modules (head, middle, and tail), thereby integrating the overall structure and enabling effective inter-module communication [13-16]. Structurally, Med14 extends across the Mediator like a backbone or spine, physically linking the modules together. In *S. pombe*, cryo-EM studies revealed that the absence of Med14 causes the entire Mediator complex to disassemble, underscoring its indispensable role as the architectural framework [6, 16]. Med17, also part of the head module, plays a scaffold-like role for the assembly and stabilization of the head module and facilitates its interaction with the middle module [8, 13, 14, 16, 17]. High-resolution structures demonstrate that Med17 serves as the hub of the head, anchoring other subunits, forming the fixed jaw domain and establishing a docking interface with the middle modules [18]. The absence of Med17 destabilizes the head module and prevents efficient communication with RNA polymerase II [19].

The importance of Mediator in cell cycle completion is highlighted by the phenotypes associated with mutations in its specific subunits. Mutations or deletions in the components of the head module, such as *med8^ts^*, *med17Δ50*, *med18Δ*, and *med20Δ*, result in characteristic hyphal or filamentous growth due to severe defects in cell separation [7, 20]. In contrast, mutations in other Mediator modules, such as *med12Δ* of the Cdk8 modules, primarily affect cell adhesion, but not cell separation [7]. The observations suggested a specialized role of the Mediator head domain in controlling cell cycle, particularly during the final stages of cell separation.

Among the cell cycle-regulatory transcription factors in *S. pombe*, Ace2 plays a central role in cytokinesis and cell separation. It is a C2H2-zinc finger transcription factor that activates genes involved in septum degradation and cell wall remodeling. Its expression is tightly regulated by the cell cycle and peaks during the M-G1 transition [21, 22]. The regulation is linked to upstream factors, such as Sep1 and the PBF complex, which display periodic DNA-binding activity during the cell cycle [22-24]. Notably, the deletion of Ace2 results in severe cell separation defects, including the formation of elongated branched cell chains, which are phenotypes observed in Mediator head-domain mutants as well [7, 25]. Although a connection between the Mediator complex and Ace2-dependent cell separation has already been proposed, the precise molecular mechanisms by which the individual Mediator subunits contribute to Ace2 function remain unclear.

In this study, we compared the roles of three key Mediator subunits, namely Med8, Med14, and Med17, using a genome-wide approach involving RNA-seq and ChIP-seq analyses. Our findings demonstrate that Med8 plays a distinct and critical role in supporting Ace2 chromatin binding and target gene expression. The results revealed a subunit-specific regulatory mechanism within the Mediator complex that facilitates cell cycle-dependent transcription in *S. pombe*. Furthermore, our study expanded the understanding of how the Mediator subunit can precisely coordinate transcription factor activity to ensure accurate cell division.

## Materials and Methods

### Yeast Strains and Cell Culture

All strains were constructed based on a wild-type strain using standard genetic modifications. For all experiments, yeast strains were cultured in Yeast Extract Supplemented medium at 30°C. To employ the auxin inducible system, cells were treated with 100 nM 5-admantyl-IAA (5-Ad-IAA) at 30°C for 30 min, and the control was treated with the same volume of DMSO. To synchronize mitosis, cell cycle arrest was induced by treatment with 12 mM hydroxyurea for 4 h, which arrested the cells in early S-phase. Next, the cells were washed twice and released into a fresh Yeast Extract Supplemented medium for 100 min.

### 4',6-Diamidino-2-Phenylindole Staining

Ten milliliters of cells were grown to mid-exponential phase (OD_600_ = 0.5), fixed with 1% glutaraldehyde on ice for 20 min, and washed twice with PBS. Fixed cells were stained with 1 μg of 4',6-diamidino-2-phenylindole in PBS for 5 min at room temperature in the dark. After washing with PBS, the cells were resuspended in PBS and observed under a fluorescence microscope.

### RNA Isolation and qRT-PCR

Twenty milliliters of cells were grown to mid-exponential phase (OD_600_ = 0.5). The cells were treated with 5-Ad-IAA for 30 min and harvested by centrifugation at 3,000 rpm for 5 min at 4°C. Total RNA was extracted using an RNeasy Mini Kit (Qiagen, 74104, Germany) according to the manufacturer’s instructions. Subsequently, DNA was removed from the purified RNA using a TURBO DNA-free kit (Invitrogen, AM1907, USA), according to the manufacturer’s instructions. RNA concentration and purity were measured using a Qubit fluorometer (Q33226; Thermo Fisher Scientific, USA). For cDNA synthesis, 1 μg of total RNA was reverse-transcribed using random hexamers and RevertAid reverse transcriptase (Thermo Fisher Scientific, EP0442). Quantitative real-time PCR was performed using primers designed specifically for the target genes.

### RNA-seq and Data Analysis

For library preparation, 1 μg of total RNA was used for mRNA isolation. mRNA was isolated using NEBNext Poly(A) mRNA Magnetic Isolation (NEB, E7490), following the manufacturer’s instructions. Library preparation was performed using the NEBNext Ultra RNA Library Prep Kit for Illumina (NEB, E7770, USA) and NEBNext Multiplex Oligos for Illumina (Index Primer Set 1) (NEB, E7335S) according to the manufacturer’s instructions. The libraries were sequenced using an Illumina NovaSeq 6000 system (Macrogen, Republic of Korea). The raw sequencing data were trimmed and aligned to the fission yeast reference genome. Gene expression levels were quantified in terms of reads per kilobase million (RPKM) values. Differentially expressed genes were analyzed using the DESeq2 R package (version 1.44.0).

### Chromatin Immunoprecipitation (ChIP) and qPCR

ChIP was performed with modifications of the previously published protocol [26]. Fifty milliliters of cells were grown to mid-exponential phase (OD_600_ = 0.5) and fixed with 3% paraformaldehyde at 18°C for 30 min. Secondary fixation was performed with dimethyl adipimidate dihydrochloride at room temperature for 45 min. Dyna-G beads (Thermo Fisher Scientific, 10004D) were pre-incubated at 4°C with either anti-DDDDK-tag (MBL, M185-3L), anti-Myc (MBL, M047-3), or anti-RNA polymerase II CTD (Abcam, ab26721, United Kingdom) antibodies. Then, the fixed cells were resuspended in 400 μl of ChIP Lysis Buffer (50 mM HEPES-KOH pH 7.5, 140 mM NaCl, 1 mM EDTA, 1% Triton X-100, 0.1% DOC, 1 mM PMSF, cOmplete Protease Inhibitor). Cells were disrupted with glass beads using a bead beater. Next, DNA was sheared with a Bioruptor Pico sonicator. The collected lysates were precleared with Dynabeads Protein G and immunoprecipitated with antibody-bound beads. After immuno-precipitation, beads were washed twice with lysis buffer, once with high-salt lysis buffer, once with wash buffer, and once with Tris-EDTA (TE) buffer. The DNA bound to the beads was eluted, followed by reverse-crosslinking overnight at 65°C, and treated with proteinase K. IP DNA was purified using the QIAquick PCR Purification Kit (Qiagen, 28104), and its concentration was quantified using a Qubit fluorometer (Thermo Fisher Scientific, Q33226). For quantitative PCR, IP DNA and input DNA were diluted, and primers were designed to specifically target the protein-binding regions.

### ChIP-seq and Data Analysis

For library preparation, total immunoprecipitated DNA was processed using the NEBNext Ultra II DNA Library Prep Kit (NEB, E7770). Subsequent steps followed the methods described in the RNA-seq and data analysis section. Raw data were trimmed and mapped to the reference genome using Bowtie2. Enrichment levels were quantified using transcripts per million (TPM) and visualized using IGV (Integrative Genomics Viewer). Gene Ontology (GO) term analysis of the occupied gene lists was performed using the AnGeLi software. Additionally, a correlation analysis between Med14 and Pol II was conducted using deepTools (version 3.5.5).

### Western Blotting

Fifty milliliters of cells were grown to mid-exponential phase (OD_600_ = 0.5) and harvested by centrifugation at 3,000 rpm for 5 min at 4°C. Cell pellets were resuspended in 500 μl phosphate buffer with 1 mM PMSF and Complete Protease Inhibitor. Cells were disrupted with glass beads using a bead beater. Crude lysates were collected by centrifugation at 14,000 rpm for 15 min at 4°C. Protein concentrations were determined using Qubit fluorometer. The samples were normalized, mixed with 4× Laemmli sample buffer, and boiled for 5 min. Equivalent amounts of protein were loaded onto 10% SDS-PAGE gels and subsequently transferred to nitrocellulose membranes. Membranes were blocked and incubated with primary antibody at 4°C overnight using anti-mini-AID (1:500, MBL, M214-3), anti-Myc (1:2500, MBL, M047-3), anti-FLAG (1:5,000, MBL, M185-3L), and anti-β-tubulin (1:5000, BioAcademia, 63-160). After three TBST washes, the membranes were incubated with horseradish peroxidase-conjugated secondary antibodies (1:10,000, Bio-Rad, BR170-6516, BR170-6515) for 1–2 h at room temperature. Signals were detected using chemiluminescence and visualized using an e-Blot imaging system.

### Co-Immunoprecipitation (Co-IP)

Co-IP was performed according to a previously published protocol, with modifications [26]. Dyna-G beads (Thermo Fisher Scientific, 10004D) were pre-incubated at 4°C for 2 h with either anti-DDDDK tag (MBL, M185-3L) or anti-PK (Bio-Rad, MCA1360, USA). Fifty milliliters of cells were grown to mid-exponential phase (OD_600_ = 0.5) and harvested at 3,000 rpm at 4°C. Cell pellets were resuspended in 500 μl of IP buffer (50 mM HEPES, 7.6, 75 mM KCl, 0.1% NP-40, 20% Glycerol, 1 mM EDTA, 1 mM PMSF, cOmplete Protease Inhibitor). Cells were disrupted with glass beads using a bead beater. Crude lysates were collected by centrifugation at 14,000 rpm for 15 min at 4°C. The lysates were precleared with Dyna-G beads for 30 min at 4°C to reduce nonspecific binding. After pre-clearing, 0.5% of the total lysate was aliquoted as the input. The remaining lysate was incubated overnight at 4°C with antibody-bound DynaG beads. After incubation, the beads were washed six times with IP buffer for 5 min for each wash and boiled with 4× Laemmli sample buffer for elution. The subsequent western blotting steps were identical to those described previously, except for the secondary antibody (1:5000, Abcam, ab131368).

## Results

### Rapid Depletion of Essential Mediator Subunits Using the Auxin-Inducible Degron (AID) System

**Fig. 1:**
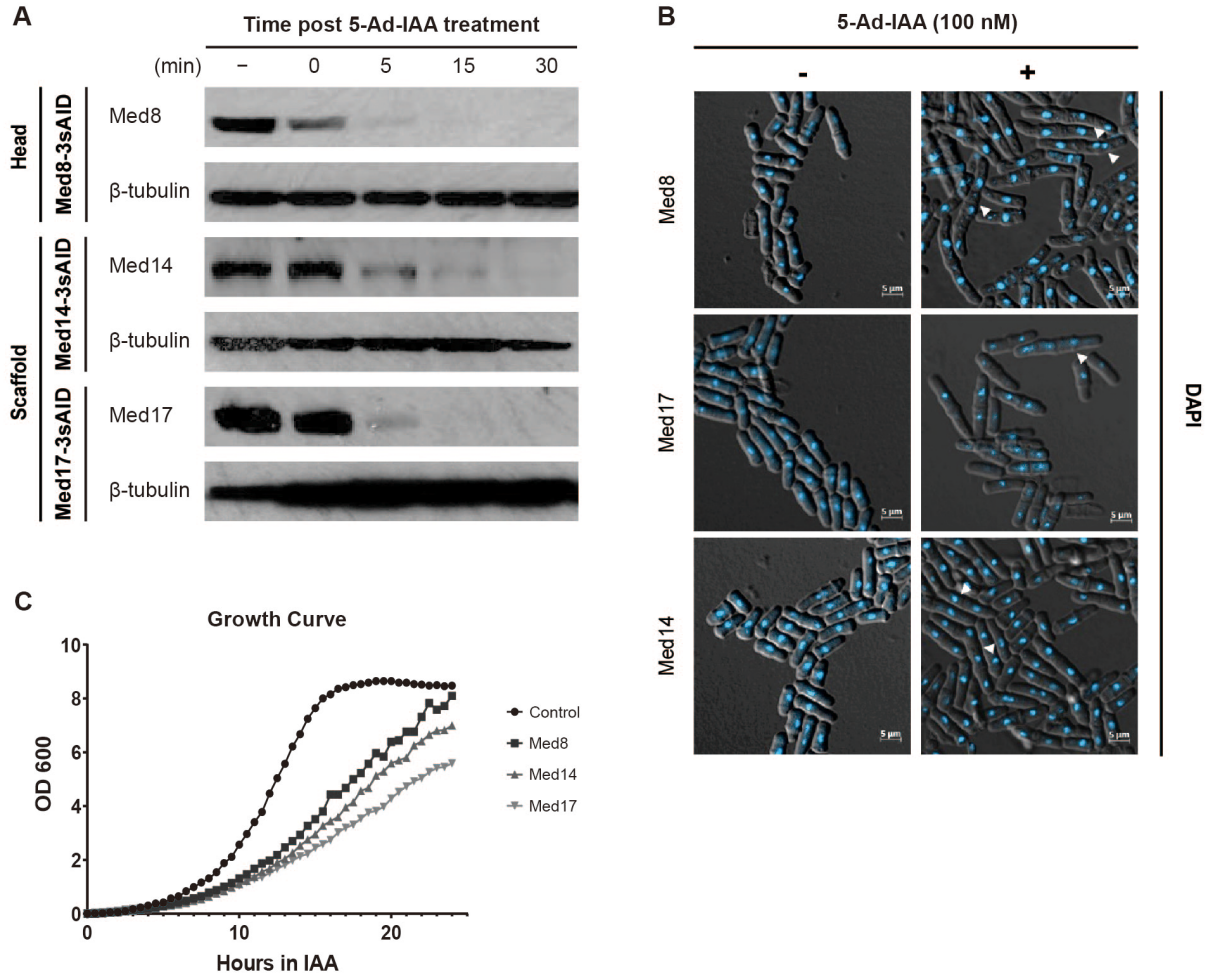
Depletion of Mediator subunits with the AID system. (**A**) Western blot analysis of AID-tagged Mediator subunits Med8 (head), Med17 (scaffold), and Med14 (scaffold). Samples were collected at the indicated time points (0, 5, 15, and 30 min) following treatment with 100 nM 5-Admantyl-IAA (5-Ad-IAA). Mediator subunits were detected using antimini- AID antibody. β-tubulin served as a loading control. (**B**) Phenotypic analysis of AID-tagged Mediator mutants. Abnormal cell separation was observed in samples treated with or without 100 nM 5-Ad-IAA. Abnormally separated cells are indicated by white arrows. Nuclei were visualized by 4',6-diamidino-2-phenylindole staining. (**C**) Growth curves of AIDtagged Mediator mutants following treatment with 100 nM 5-Ad-IAA compared to those of DMSO-treated control samples. OD_600_ measurements were recorded over a 24-h period.

We utilized the AID system to achieve acute depletion of the essential Mediator subunits (Med8, Med14, and Med17). Each subunit was fused C-terminally with an AID degron to generate conditional mutant strains. Upon addition of 5-Ad-IAA, all AID-tagged proteins were rapidly and efficiently degraded. The depletion of each subunit was nearly complete within 30 min of auxin addition, demonstrating the effectiveness of this system in acute protein depletion (Fig. 1A).

Following depletion, all three mutant strains exhibited pronounced abnormal hyphal-like growth characterized by defective cell separation and elongated morphology (Fig. 1B). The phenotypes indicated that Med8, Med14, and Med17 are required for proper cell division and maintenance of normal morphology. When we monitored cell growth in liquid culture, depletion of Med8, Med14, or Med17 significantly impaired cell growth than in the DMSO-treated control. Notably, the loss of Med14 or Med17 resulted in more severe growth defects than the loss of Med8 (Fig. 1C). This was in line with previous findings that Med14 and Med17 play more central roles within the Mediator by bridging other modules and maintaining the integrity of the complex [8, 14-16, 27].

Taken together, the results demonstrated that acute depletion of essential Mediator subunits via the AID system leads to profound defects in both cell morphology and proliferation, reflecting the essential role of each Mediator subunit in maintaining cellular function and viability.

### Med8 Specially Regulates the Transcription of Ace2 Target Genes

**Fig. 2:**
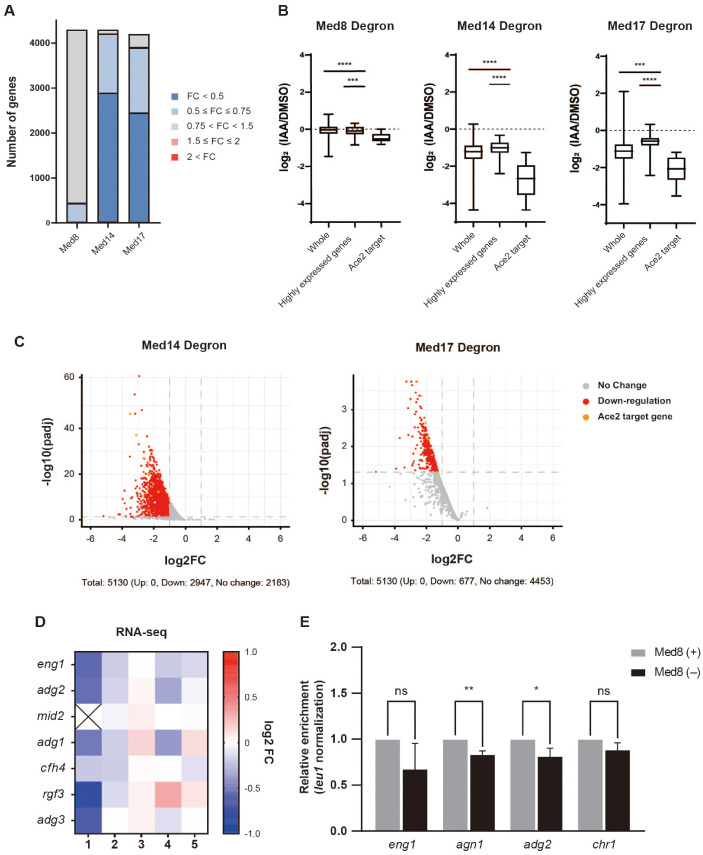
Effect of Med8, Med14, and Med17 depletion on global transcriptional regulation. (**A**) Bar graph showing the number of differentially expressed genes upon depletion of Med8, Med14, or Med17, as determined by RNA-seq. Genes were categorized based on fold changes (FC) in RPKM values (IAA vs. DMSO treatment). (**B**) Box plots showing log_2_ fold changes (IAA/DMSO) in RNA-seq signals for each auxin-treated AID-tagged mutant strain, categorized into all genes (whole), highly expressed genes, and Ace2 target genes. Statistical analysis was performed using the Mann–Whitney U test. (**C**) Volcano plots showing transcriptomic changes in Med14 and Med17 degron mutants following IAA treatment. Horizontal dashed lines represent the adjusted p-value threshold (< 0.05), and vertical dashed lines indicate ± log_2_(fold-change) cutoffs. Genes with more than two-fold downregulation are shown in red; Ace2 target genes are highlighted in orange. (**D**) Heatmap showing log_2_ (fold changes) in the expression of Ace2 target genes across five biological replicates of RNA-seq from the Med8 degron mutant. Red indicates upregulation, blue indicates downregulation relative to DMSO-treated controls. (**E**) Quantitative RT-PCR analysis of Ace2 target gene expression (*eng1*, *agn1*, *adg2*, and *chr1*) in Med8 degron cells treated with IAA or DMSO. Expression levels were normalized with respect to a control gene. Statistical significance was determined using an unpaired *t*-test. **p < 0.01, *p < 0.05, ns = not significant.

To investigate how the depletion of individual Mediator subunits affects global transcriptional regulation, we performed RNA-seq analysis of the degron-tagged strains. Each strain was treated with 100 nM 5-Ad-IAA for 30 min to induce rapid degradation of the target protein, and the resulting transcriptome profiles were compared with those of the DMSO-treated controls. Differentially expressed genes were categorized based on the fold-change in RPKM values (Fig. 2A). Depletion of Med14 or Med17 led to global downregulation of gene expression, affecting 4,210 or 3,896 genes, respectively. In contrast, the depletion of Med8 resulted in a much smaller transcriptional impact, with only 436 genes (approximately 10% of the genome) showing significant downregulation (Fig. 2A).

Further stratification of the gene expression changes revealed context-specific regulatory roles. In the Med8-depleted strain, overall transcriptomic alterations were minimal, both across the entire transcriptome and among the highly expressed gene groups. Nevertheless, a notable decrease in the expression of Ace2 target genes was observed, suggesting that Med8 plays a more specialized role in the transcriptional regulation of this gene set (Fig. 2B). In contrast, depletion of Med14 or Med17 led to broad and substantial transcriptional repression across all gene categories examined, including the whole transcriptome, highly expressed genes, and Ace2 targets (Fig. 2B). The downregulation of highly expressed genes was comparable to that of the whole transcriptome, suggesting that Mediator dysfunction did not affect the actively expressed genes disproportionately (Fig. 2B). Notably, the Ace2 target genes were among the most severely repressed, exhibiting a greater than four-fold reduction, underscoring their strong dependence on Mediator activity.

To validate such transcriptional trends, volcano plot analyses were performed using the RNA-seq data from two biological replicates of the Med14 and Med17 degron strains. The plots confirmed that many genes were strongly downregulated in the absence of either subunit; particularly, Ace2 target genes were among the most significantly affected (Fig. 2C). Due to poor reproducibility between replicates, volcano plot analysis of the Med8 degron strains was not possible. Instead, we specifically focused on the expression trends of Ace2 targets. Heatmap analysis of five independent Med8-depleted samples consistently showed a downward trend in the expression of Ace2-regulated genes (Fig. 2D). The observations were further supported by qRT-PCR, which confirmed significant reductions in the relative mRNA levels of representative Ace2 target genes upon Med8 depletion (Fig. 2E).

In summary, Med14 and Med17 were broadly required for transcriptional activity across the genome, whereas Med8 appeared to play a more specialized role, particularly in the expression of Ace2 target genes. The findings illustrated the differential contributions of individual Mediator subunits to gene regulation and underscored the modular functionality of the Mediator complex in eukaryotic transcription.

### Mediator Binding Sites Correlate with the Highly Transcribed Genes and Ace2 Targets

Next, we investigated the genome-wide distribution of Mediator subunits using ChIP-seq. Recognizing the Mediator's established role in PIC assembly, we focused our analysis on the transcription start sites (TSSs), where the Mediator typically binds [28, 29]. Accordingly, a list of Mediator-binding genes was generated based on a ChIP-seq signal (expressed as TPM) exceeding 250 within the TSS±500-bp region.

**Fig. 3:**
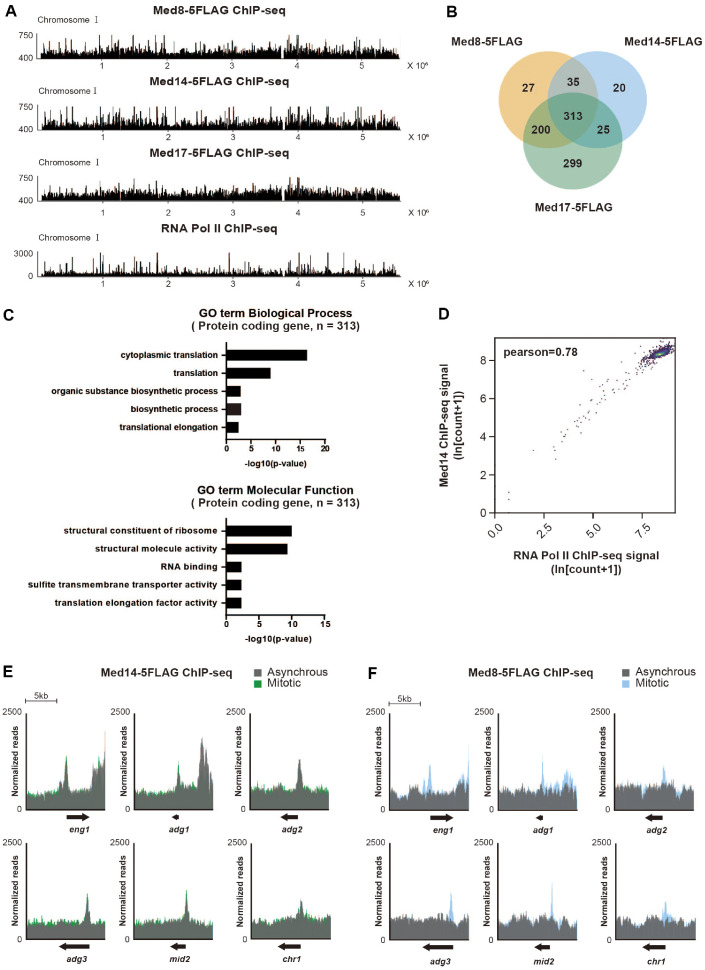
Genome-wide association of Mediator components. (**A**) ChIP-seq profiles showing genome-wide binding of Mediator subunits Med8-5FLAG, Med14-5FLAG, and Med17-5FLAG, as well as RNA Polymerase II (RNA Pol II), on chromosome I from asynchronously growing cells. (**B**) Venn diagram showing the overlap of genes bound by Med8, Med14, and Med17. Bound genes were defined as those with ChIP-seq signal (TPM > 250) within ± 500 bp of the transcription start sites (TSS). (**C**) Gene ontology (GO) enrichment analysis (Biological Process and Molecular Function) of the 313 genes cooccupied by Med8, Med14, and Med17. (**D**) Scatter plot showing the correlation between Med14-5FLAG and RNA Pol II genome-wide occupancy based on ChIP-seq signal intensities. A positive correlation was observed (Pearson r = 0.78). (**E**, **F**) ChIP-seq profiles of Med14-5FLAG (**E**) and Med8-5FLAG (**F**) at Ace2 target genes (*eng1*, *adg1*, *adg2*, *adg3*, *mid2*, and *chr1*) in asynchronous growing cells (gray) and cells synchronized in mitosis (blue for Med8, green for Med14).

Genome-wide binding profiles revealed that Med8-5FLAG, Med14-5FLAG, and Med17-5FLAG were broadly distributed across all chromosomes (Fig. 3A). We identified 575, 393, and 837 binding sites for Med8-5FLAG, Med14-5FLAG, and Med17-5FLAG, respectively. Venn diagram analysis revealed a high degree of shared occupancy among the three subunits, indicating substantial co-binding (Fig. 3B). Although 299 genes appeared to be exclusively bound by Med17-5FLAG, the signals were likely background noise due to elevated ChIP-seq background levels.

To characterize the common binding sites of the Mediator complex, we performed GO term analysis of the 313 genes co-occupied by all three Mediator subunits. The analysis revealed significant enrichment of genes associated with fundamental cellular processes. Notably, among the “Biological Process” terms, translation and organic substance biosynthetic process were prominently represented, whereas “Molecular Function” terms were enriched for structural constituent of ribosome, structural molecule activity, and RNA binding (Fig. 3C). The prevalence of these terms suggested that the Mediator preferentially associates with genes involved in essential and highly active cellular processes, particularly those related to protein synthesis and ribosome assembly.

To test whether Mediator binding correlates with RNA Pol II, we conducted a ChIP-seq analysis of RNA Pol II and found that its genome-wide binding profile substantially overlapped with that of the Mediator subunits (Fig. 3A). Genome-wide correlation analysis between Med14-5FLAG and RNA Pol II revealed a strong positive correlation (Pearson’s correlation coefficient (r) = 0.78), supporting the notion that Med14, and by extension, the entire Mediator complex, preferentially targets actively transcribed genes (Fig. 3D). However, direct evidence of Mediator-chromatin binding regulating mRNA transcription remains inconclusive, since the depletion of Mediator subunits Med14 and Med17 did not specifically affect the expression levels of the highly expressed genes in our RNA-seq analysis (Fig. 2B), despite the strong genome-wide correlation between Mediator occupancy and RNA Pol II binding (Fig. 3D).

Following our observation that Med8 plays a specific and critical role in the transcriptional regulation of Ace2 target genes (Fig. 2), we examined the chromatin binding of Mediator subunits at these loci. Notably, Med14-5FLAG exhibited consistently pronounced enrichment in the TSS regions of the Ace2 target genes across both the asynchronous and mitotic phases (Fig. 3E). In contrast, Med8-5FLAG showed barely detectable peaks in the asynchronous phase, with a distinct and prominent peak exclusively during the mitotic phase (Fig. 3F). The result suggested that, unlike Med14, Med8 binding at Ace2 target loci is much weaker and can only be clearly detected when the population is enriched in mitotic cells.

### Med8 Depletion Does Not Affect the Chromatin Binding of Core Mediator Subunits Med14 and Med17

**Fig. 4:**
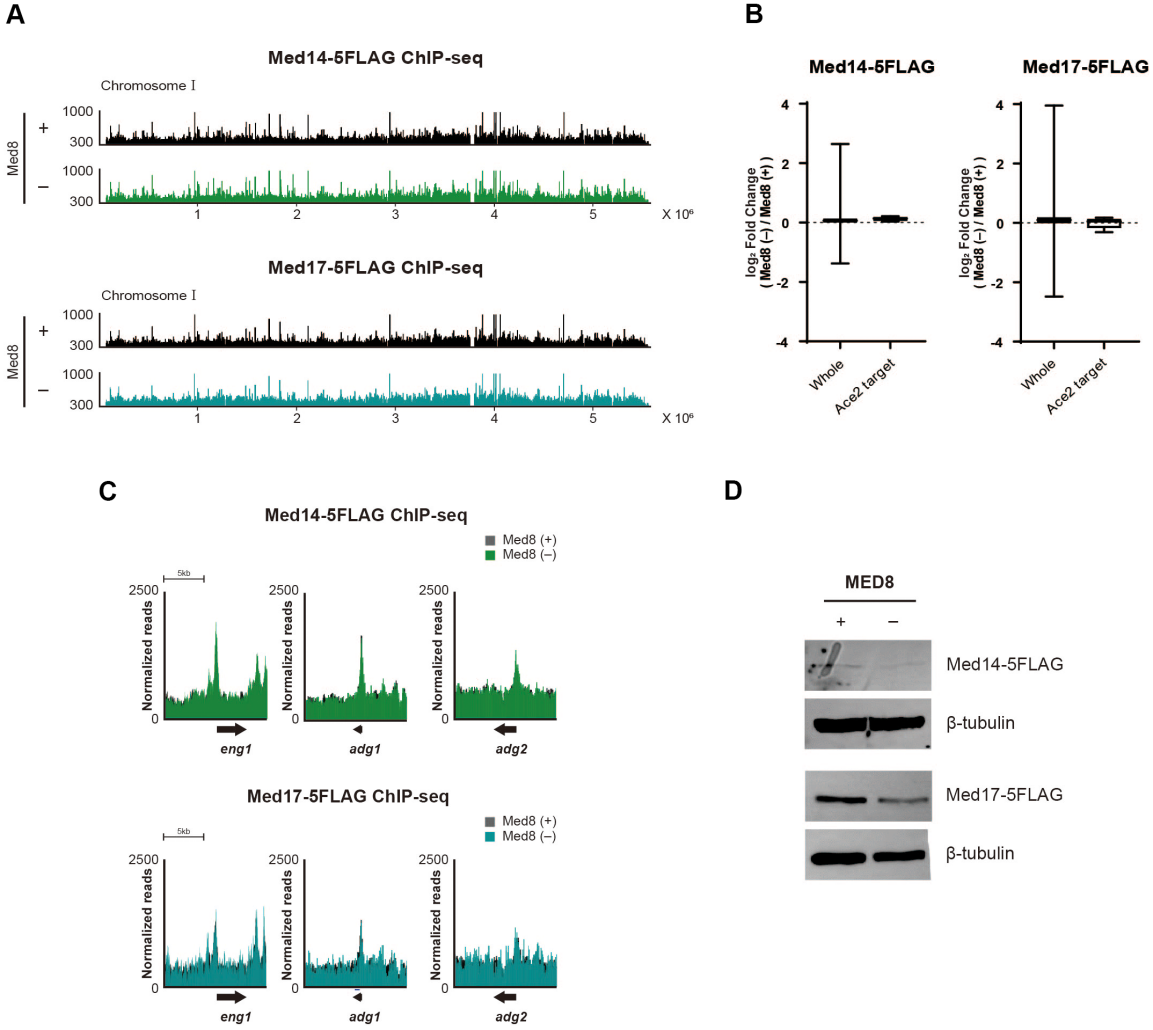
ChIP enrichment of Med14 and Med17 at Ace2 target genes in asynchronous cells upon Med8 depletion. (**A**) ChIP-seq profiles illustrating the genome-wide binding of Med14-5FLAG (top) and Med17-5FLAG (bottom) on chromosome I , with and without Med8. (**B**) Box plots depicting log_2_ fold changes in ChIP-seq signal (as TPM) for Med14- 5FLAG and Med17-5FLAG under Med8-depleted versus Med8-intact conditions. Genes were categorized into whole genes and Ace2 target genes. (**C**) ChIP-seq profiles for Med14-5FLAG (top) and Med17-5FLAG (bottom) at representative Ace2 target genes (*eng1*, *adg1*, and *adg2*). (**D**) Western blot analysis showing the protein levels of Med14 and Med17 under conditions with or without Med8. β-tubulin served as a loading control.

To investigate the role of Med8 in the genomic binding of the Mediator complex, including Ace2 target genes, we performed ChIP-seq analysis of two other essential Mediator subunits, Med14 and Med17, with and without Med8. The genome-wide binding profiles of Med14-5FLAG and Med17-5FLAG remained largely unchanged following Med8 depletion (Fig. 4A). Consistently, the log_2_ fold changes in ChIP-seq signals (as TPM) showed minimal or negligible differences across the genome and at Ace2 target genes under Med8-depleted conditions (Fig. 4B). Furthermore, the binding of Med14 and Med17 to the representative Ace2 target genes *eng1*, *adg1*, and *adg2*, was unaffected in the absence of Med8 (Fig. 4B and 4C). The results suggested that although Med8 contributes specifically to the transcriptional regulation of Ace2 target genes, its absence does not impair the recruitment of other core Mediator subunits to these target loci. In addition, Med14 and Med17 protein levels remained largely stable regardless of Med8 expression, with only negligible changes observed.

### Ace2 Binding to Its Target Genes Is Reduced in the Absence of Med8

In our previous study, we reported that Med8 regulates the expression of Ace2 target genes (Fig. 2). However, the regulatory effect was not strongly associated with changes in Mediator complex binding (Fig. 4). Therefore, we hypothesized that Med8 influences the expression of Ace2 target genes by modulating the chromatin binding of Ace2. To test this hypothesis, we performed ChIP-seq analysis of Ace2 under conditions in which individual Mediator subunits were depleted.

**Fig. 5:**
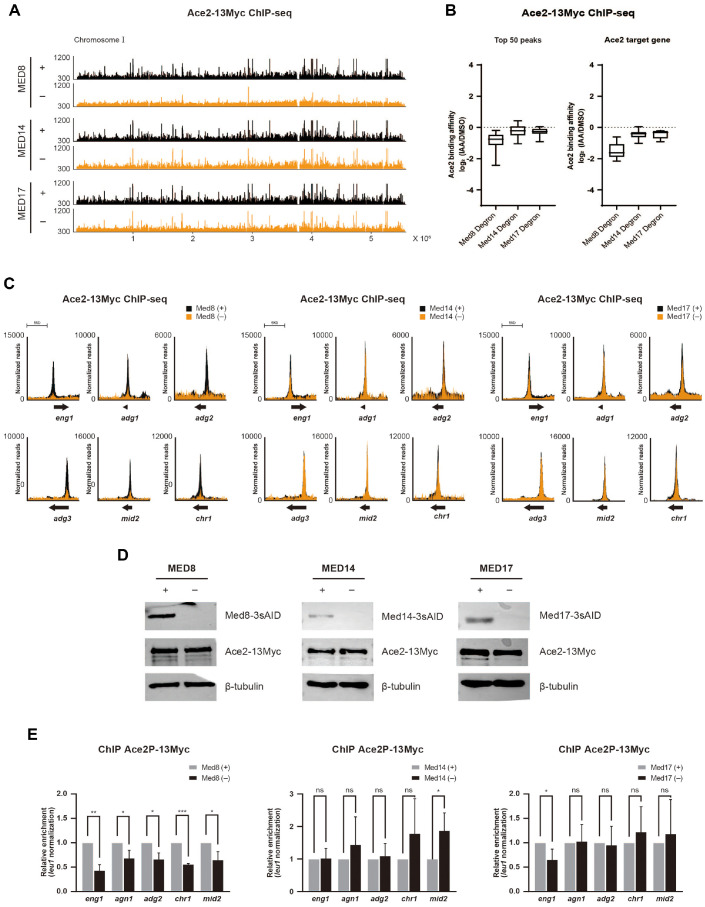
ChIP enrichment of Ace2 at its target genes upon depletion of Mediator subunits. (**A**) ChIP-seq profiles displaying genome-wide binding of Ace2-13Myc on chromosome I under control conditions and following depletion of individual Mediator subunits (Med8, Med14, or Med17) from asynchronously growing cells. (**B**) Box plots showing log_2_ fold changes in Ace2-13Myc ChIP-seq signal (as TPM) after depletion of Med8, Med14, or Med17. The left panel represents the top 50 Ace2-binding peaks with the highest occupancy; the right panel shows the Ace2 target genes (n = 8) known to be transcriptionally regulated by Ace2. (**C**) ChIP-seq analysis of Ace2-13Myc occupancy at representative Ace2 target genes (*eng1, adg1, adg2, adg3, mid2*, and *chr1*) following depletion of each Mediator subunit. (**D**) Western blot analysis showing the protein levels of Ace2 under Med8, Med14, or Med17 depletion using asynchronous cells. β-tubulin served as a loading control. (**E**) ChIP-qPCR analysis of Ace2 binding at Ace2 target genes during mitosis, under control and Mediator subunit-depleted conditions. Statistical significance was determined using an unpaired t-test: ***p < 0.001, **p < 0.01, *p < 0.05, ns = not significant.

The loss of Med8 resulted in a remarkable reduction in Ace2 occupancy across the entire genome. Although the depletion of Med14 and Med17 led to a modest decrease in Ace2 binding, the effect was substantially weaker than that of Med8 depletion (Fig. 5A). To quantify the observations, we calculated the log_2_ fold changes in the Ace2 ChIP-seq signals (as TPM) under each depletion condition. Analysis of the top 50 Ace2-binding peaks representing regions with high Ace2 occupancy revealed a consistent reduction in Ace2 binding under all three depletion conditions, with the strongest effect observed upon Med8 depletion (Fig. 5B, left panel). Similarly, Ace2 occupancy at eight known Ace2 target genes was significantly reduced in all depletion backgrounds, with the most pronounced effect observed in Med8-depleted cells (Fig. 5B, right panel). We further examined Ace2 occupancy at individual representative Ace2 target genes, including *eng1*, *adg1*, *adg3*, *mid2*, and *chr1*. Consistent with genome-wide results, Ace2 binding at these loci was significantly diminished by Med8 depletion (Fig. 5C).

To exclude the possibility that the binding affinity of Ace2 was due to changes in protein expression levels, we analyzed Ace2 protein levels in the presence or absence of each Mediator subunit. Western blot analysis revealed no significant difference in Ace2 expression, indicating that the observed impairment in chromatin binding was not due to Ace2 unavailability, but rather due to its compromised recruitment to target loci (Fig. 5D).

Ace2 is a key transcription factor that is required for cell separation during the final stage of mitosis. To assess whether the Mediator specifically influences Ace2 chromatin occupancy during mitosis, we performed a ChIP-qPCR analysis of Ace2 binding to its target genes in synchronized mitotic cells under Mediator-subunit depletion conditions. Consistent with ChIP-seq data from asynchronously growing cells, Med8 depletion significantly reduced Ace2 binding during mitosis. In contrast, the depletion of Med14 and Med17 had little to no effect on Ace2 occupancy under the same conditions (Fig. 5E).

Together, the findings demonstrated that Mediator subunits, particularly Med8, are essential for the stable chromatin recruitment of Ace2, thereby playing a central role in the precise transcriptional regulation of genes involved in cell separation during mitosis. Thus, Med8 is a critical co-regulator of Ace2 function, ensuring its binding to the target genes necessary for proper execution of the mitotic exit program.

### Med8 and Ace2 Interact Physically

**Fig. 6:**
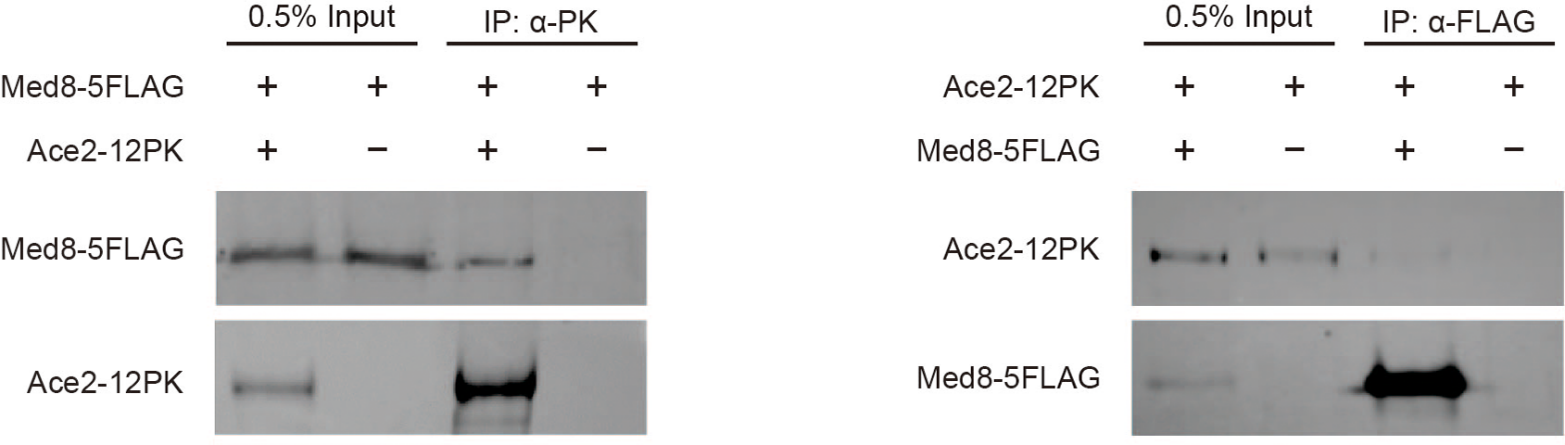
Physical interaction between Med8 and Ace2. Co-immunoprecipitation (co-IP) analysis revealed a physical interaction between Ace2-12PK and Med8-5FLAG. Immunoprecipitation was performed using either an anti-PK antibody (left) to pull down Ace2-12PK or an anti-FLAG antibody (right) to pull down Med8-5FLAG. The presence of Med8-5FLAG in Ace2-12PK and Ace2-12PK in Med8-5FLAG immunoprecipitates was detected by immunoblotting with anti-FLAG and anti-PK antibodies, respectively.

To investigate the molecular mechanisms underlying the specific regulation of Ace2 target genes, we examined whether Ace2 interacted physically with Med8. We performed a co-IP analysis to assess the potential physical association between Ace2 and Med8 (Fig. 6). Results revealed that Ace2 indeed interacts with Med8. Specifically, Med8 was detected in the Ace2 immunoprecipitates using an anti-PK antibody, and reciprocally, Ace2 was detected in the Med8 immunoprecipitates using an anti-FLAG antibody. The reciprocal co-IP results provided strong evidence for a direct or closely associated physical interaction between the transcription factor Ace2 and the Mediator subunit Med8. However, further experiments will be required to determine the precise nature of this association.

## Discussion

Our study revealed the distinct roles of the Mediator subunits Med8, Med14, and Med17 in the regulation of Ace2-dependent gene transcription in *S. pombe*, with a particular focus on Med8 as a specific regulator. Unlike Med14 and Med17, Med8 was found to exert a direct regulatory effect by facilitating the initiation of Ace2-dependent transcription.

The central role of Med8 was underscored by the severe separation abnormalities and cell growth defects observed upon its depletion. Transcriptome analysis under these conditions demonstrated remarkable downregulation of a selected group of genes, notably those regulated by Ace2. Genome-wide ChIP-seq further confirmed a dramatic reduction in Ace2 occupancy at its target promoters despite unchanged Ace2 protein levels. The results enhanced our understanding of Med8 as a coactivator within the Sep1-Ace2 transcriptional cascade [22, 23, 30], emphasizing its essential role in the initial recruitment phase.

Med8 depletion did not affect the expression or genomic occupancy of other Mediator subunits, such as Med14 and Med17, indicating that its role is functionally distinct and not due to the general destabilization of the Mediator complex. In support of a direct interaction, a physical association between Med8 and Ace2 was confirmed, reinforcing the specific coactivator function of Med8 in Ace2-mediated transcription. The findings extended the current understanding by highlighting how the individual Mediator subunit Med8 engages directly with the specific transcription factor Ace2 to fine-tune transcriptional programs.

In contrast, Med14 and Med17 primarily served as integral architectural scaffolds. Their depletion destabilized the entire Mediator complex and led to broad transcriptional downregulation, including that of Ace2 target genes. Our observation that Med14 or Med17 depletion leads to widespread transcriptional downregulation is strongly consistent with the previous findings in *Saccharomyces cerevisiae* [19]. However, crucially and distinct from the impact of Med8, the binding of Ace2 to its target genes remained largely unperturbed by the depletion of Med14 or Med17, indicating that Med14 and Med17 are critical not for the recruitment, but for post-recruitment transcriptional activation. Our data suggested that even when Ace2 is properly localized to its promoter targets, efficient transcription fails in the absence of a stable Mediator framework, likely due to impaired conformational changes in the defective signal transduction necessary for RNA polymerase II engagement and promoter escape.

In addition, genome-wide mapping revealed that Med8, Med14, and Med17 largely co-localized at the same loci, exhibiting binding patterns highly similar to those of RNA Pol II. The pattern reflected the conserved nature of Mediator functions across eukaryotes [31]. While Mediator dynamics and recruitment patterns have been extensively studied in *S. cerevisiae*, particularly in the upstream activating sequences and PICs [28, 32, 33], systematic genome-wide analyses in yeast, especially at the level of individual subunits and specific cell cycle phases, remain limited.

Mediator-dependent transcriptional regulation is enabled through a diverse and modular network of interactions between individual Mediator subunits and transcription factors (TFs), the complexity of which increases with eukaryotic evolution [13, 34, 35]. While early studies in yeast identified the tail module, particularly Med15, as the primary interface for activator binding, subsequent research in higher eukaryotes has shown that TFs can engage multiple subunits across all core Mediator modules, including the head, middle, and kinase modules [13, 35, 36]. This expanded landscape of TF-Mediator contacts supports highly subunit-specific regulatory mechanisms, enabling the integration of a wide spectrum of gene regulatory signals. In this context, further investigation into the distinct roles of each Mediator module, including the head, middle, tail, and Cdk8 kinase modules, in *S. pombe* would provide valuable insights into the modular and context-dependent functions of the Mediator in transcriptional regulation. Additionally, in contrast to the specialized function of Med8 with Ace2 in our study, the role of Med8 in *Saccharomyces cerevisiae* has been primarily studied in the perspective of its contribution to the entire Mediator complex and its broad participation in transcription initiation [9, 33]. These suggest that, while the structural role of Med8 within the Mediator head is conserved, its functional specialization regarding specific transcription factors, such as Ace2, might be species-specific, suggesting the evolutionary diversity of Mediator-TF interactions.

In conclusion, our study comprehensively elucidated the distinct functional roles of the Mediator complex and its individual subunits, particularly Med8, in contributing to the precise molecular mechanisms regulating the function of the specific transcription factor Ace2. Additionally, the study provided comprehensive insights into the genome-wide chromatin-binding patterns of the Mediator complex in fission yeast.
